# EFNB1 Acts as a Novel Prognosis Marker in Glioblastoma through Bioinformatics Methods and Experimental Validation

**DOI:** 10.1155/2021/4701680

**Published:** 2021-11-16

**Authors:** Yaohong Shi, Yuanyuan Sun, Hongyan Cheng, Chen Wang

**Affiliations:** ^1^Department of Neurology, The First People's Hospital of Lianyungang, The Affiliated Lianyungang Hospital of Xuzhou Medical University, The Affiliated Hospital of Kangda College of Nanjing Medical University, Lianyungang Clinical College of Nanjing Medical University, Lianyungang 222061, China; ^2^Department of Radiation Oncology, The First Affiliated Hospital of Nanjing Medical University, Nanjing 210000, China; ^3^Department of Synthetic Internal Medicine, The First Affiliated Hospital of Nanjing Medical University, Nanjing 210000, China; ^4^Department of Neurosurgery, The Affiliated Suzhou Science & Technology Town Hospital of Nanjing Medical University, Suzhou 215153, China

## Abstract

**Purpose:**

Ephrin B1 (EFNB1), the Eph-associated receptor tyrosine kinase ligand, is suggested to have an important function in neurodevelopment. However, its contribution to glioblastoma multiforme (GBM) remains uncertain. This study aimed to determine the prognostic power and immune implication of EFNB1 in GBM.

**Methods:**

We first identified differentially coexpressed genes within GBM relative to noncarcinoma samples from GEO and TCGA databases by WGCNA. The STRING online database and the maximum cluster centrality (MCC) algorithm in Cytoscape software were used to design for predicting protein-protein interactions (PPI) and calculating pivot nodes, respectively. The expression of hub genes in cancer and noncancer tissues was verified by an online tool gene expression profile interactive analysis (GEPIA). Thereafter, the TISIDB online tool with Cox correlation regression method was employed to screen for immunomodulators associated with EFNB1 and to model the risk associated with immunomodulators.

**Results:**

Altogether 201 differentially expressed genes (DEGs) were discovered. After that, 10 hub genes (CALB2, EFNB1, ENO2, EPHB4, NES, OBSCN, RAB9B, RPL23A, STMN2, and THY1) were incorporated to construct the PPI network. As revealed by survival analysis, EFNB1 upregulation predicted poor overall survival (OS) for GBM cases. Furthermore, we developed a prognostic risk signature according to the EFNB1-associated immunomodulators. Kaplan–Meier survival analysis and receiver operating characteristic method were adopted for analysis, which revealed that our signature showed favorable accuracy of prognosis prediction. Finally, EFNB1 inhibition was found to block cell proliferation and migration in GBM cells.

**Conclusion:**

The above results indicate that EFNB1 participates in cancer immunity and progression, which is the candidate biomarker for GBM.

## 1. Introduction

Glioblastoma multiforme (GBM) is a central nervous system (CNS) cancer with high malignancy grade and aggression. According to the World Health Organization (WHO) classification published in 2016, GBM is classified as a grade IV glioma [1]. Nowadays, with the advancements of medical science, in addition to surgery, chemoradiotherapy, and molecular targeted therapy, novel immunotherapy has been added to the multimode therapy for cancer. Immunotherapy is gradually gaining ground in antitumor therapy. It is well known that the discovery and application of PD-1 and its ligands PD-L1 and CTLA-4 have revolutionized the treatment of tumors [2, 3]. In recent years, multiple immunotherapies have been approved by the FDA for a variety of tumors [4]. Unfortunately, GBM patients benefit little from immunotherapy [5, 6]. This may be due to its unique anatomical location, lymphatic drainage of the CNS, blood-brain barrier, and complex tumor-immune microenvironment, which make GBM susceptible to immune escape [7, 8]. Therefore, it is extremely significant to explore the underlying pathogenesis and discover immune biomarkers to diagnose, treat, and predict the prognosis of GBM.

EFNB1 (Ephrin B1), the Eph-associated receptor tyrosine kinase ligand, mainly plays a central part in cell adhesion, angiogenesis, and development of the nervous system [9]. Abnormal expression of EFN/Eph signal in several tumors has been reported [10]. EFNB1 stimulates the secretion of matrix metalloproteinase-8, which promotes cancer cell invasion [11]. EFNB1 interacts with CNK1 to promote cell migration by activating JNK, which may have an important function in cancer metastasis [12]. Chronic hypoxia-induced slug enhances prostate cancer (PC) cell invasion and migration through upregulating EFNB1 [13]. In addition, Vermeer et al. found that EFNB1, as a PTPN13 phosphatase substrate, and its mobilization were related to ERK1/2 phosphorylation and its complex with ERBB2 mediated signal transduction and drug resistance in tumor cells [14]. At present, EFNB1 has been rarely reported in GBM, and the mechanism of its carcinogenesis remains unclear.

In our study, we constructed a gene coexpression network by analyzing the mRNA profiles obtained from TCGA and GEO databases with WGCNA analysis. Subsequently, combining differentially expressed genes (DEGs) obtained in GBM and noncarcinoma samples, we acquired differentially coexpressed genes and further built a hub gene network. EFNB1, highly expressed in GBM tissues, was screened from hub genes to construct an immunomodulator prognosis model, which could act as a novel prognosis marker and offer a new perspective for immunotherapy of GBM patients. Finally, this study examined the role of EFNB1 in carcinogenesis by in vitro study.

## 2. Methods

### 2.1. Data Collection and Processing

The original mRNA expression profiles of GBM were collected from TCGA database. Meanwhile, corresponding survival information was also obtained from TCGA data portal. Another gene profile data set of GSE108474 was obtained from the GEO database. The GSE108474 data set containing the information of 28 normal tissues and 221 tumor tissues was studied with GPL570. In addition, we obtained the clinical data of GBM cases from the Chinese Glioma Genome Atlas (CGGA) database for prognostic model validation.

### 2.2. Weighted Gene Coexpression Network Analysis (WGCNA)

The *R* software “WGCNA” package was applied to generate gene coexpression networks. First, the pickSoftThreshold function was utilized to screen soft thresholding powers to construct the scale-free networks, with TCGA data set to 3 and GSE108474 data set to 4. Second, the adjacency matrix and the topological overlap matrix (TOM) were generated based on the corresponding soft power. According to related discrepancy (1-TOM), we drew a hierarchical clustering dendrogram for classifying genes whose absolute correlation coefficients were high in the constructed coexpression modules. To determine coexpression modules related to clinical data of glioblastoma, the clinical trait information and module-trait relationships were analyzed. Finally, we selected modules with the high correlation coefficient as candidate coexpression modules for subsequent analysis.

### 2.3. Screening of DEGs and Interaction with the Modules of Interest

To search DEGs in GBM compared with noncarcinoma samples from GSE108474 and TCGA data sets, we discovered DEGs upon the thresholds of adj. *p* < 0.05 and |logFC| ≥ 1.0 by adopting the *R* software “limma” package. The volcano plot and heatmap were generated for DEGs using the *R* package “pheatmap” and “ggplot2”. Afterward, we intersected DEGs with coexpression genes obtained in the interesting modules, and the intersected genes were regarded as the candidate prognostic genes that were seen from the Venn diagram.

### 2.4. Identification of PPI Network

To predict the interaction of selected genes, we used the STRING online tool to build a PPI network. In our study, we selected genes showing the score >0.9 for network construction and visualization via Cytoscape (v3.7.2). Afterward, the maximal clique centrality (MCC) algorithm in the Cytoscape software was adopted for identifying hub genes.

### 2.5. Gene Ontology (GO) Enrichment Analysis

We conducted GO functional annotation on those screened genes by the “clusterProfiler” in the *R* package, and *p* < 0.05 was the selection criterion.

### 2.6. Validation of the Expression Patterns and the Prognostic Values of Hub Genes

The online tool GEPIA was utilized to verify hub gene expression between carcinoma and noncarcinoma tissues based on GTEx and TCGA databases. For investigating the association of OS with hub genes among GBM cases, we conducted a log-rank test and Kaplan–Meier (KM) analysis. *p* < 0.05 stood for statistical significance.

### 2.7. Immunomodulators Analysis

To explore the interactions between EFNB1 and tumor-immune system, immunomodulators correlated with EFNB1 were obtained from the TISIDB database (http://cis.hku.hk/TISIDB/). Using the TISIDB online tool, we selected immunoinhibitors and immunostimulators remarkably related to the EFNB1 level (*p* < 0.05 upon the Spearman correlation test).

### 2.8. Identification of Prognostic Model Based on EFNB1-Related Immunomodulators

First, univariate Cox regression analysis was conducted for assessing EFNB1-associated immunomodulators significantly correlated to OS based on a *p*-value of <0.05 in the training set. Then, we performed LASSO regression for narrowing the overfitting risk. At last, we carried out multivariate Cox regression analysis for constructing the optimal signature related to immunomodulators. For GBM cases, their risk scores were determined by risk score = (mRNA 1 level × coef) + (mRNA 2 level × coef) + … + (mRNA *n* level × coef). Meantime, all cases were divided into low- or high-risk group based on median score. Moreover, the CGGA data set was utilized to validate the signature.

### 2.9. Gene Set Enrichment Analysis (GSEA)

GSEA was performed to explore the downstream pathways of EFNB1. GBM samples from TCGA with the top 25% and lowest 25% EFNB1 expression were defined as the high and low expression groups, respectively. According to Molecular Signatures Database version 7.4. C1 (Hallmark), GSEA was used to obtain enriched pathways associated with EFNB1 expression.

### 2.10. Cell Culture and Transfection

We cultivated GBM cell lines (U87, U251) and normal human astrocyte line (NHA) within RPMI‐1640 medium that contained 10% fetal bovine serum (FBS, Gemini Company) under 37°C and 5% CO_2_ conditions. Negative control (si‐NC) and si‐EFNB1 were provided by RiboBio (Guangzhou, China).  Supplementary Table S1 presents the sequences of si-EFNB1. Cell transfection was performed using Lipofectamine 3000 reagent (Invitrogen) in line with specific protocols.

### 2.11. RNA Extraction and Quantitative Real-Time PCR (qRT-PCR) Assays

This study adopted TRIzol reagent (Vazyme Biotech) for extracting total cellular RNA in line with specific protocols. Later, we utilized PrimeScript reagent (Takara Bio) for preparing cDNA from RNA through reverse transcription. TB Green reagent (Takara Bio) was employed for preparing the reaction system for qRT-PCR on StepOnePlus (Thermo Fisher Scientific). Then, we measured mRNA expression based on GAPDH and determined its level through 2^−ΔΔCt^ approach.  Supplementary Table S2 displays primer sequences of all genes.

### 2.12. Cell Counting Kit-8 Assay

2000 cells per well cultured in RPMI1640 containing 10% FBS were inoculated in 96-well plates. After 24, 48, 72, and 96 h, we added 10 *μ*l CCK-8 solution (Beyotime, Shanghai, China) to incubate cells for another 2 h under 37°C in accordance with specific protocols. The spectrophotometer (Thermo Fisher Scientific) was utilized for measuring the absorbance value (450 nm).

### 2.13. Colony Formation Assay

250 cells/well were inoculated in 6-well plates separately. 10 days later, the cells grew into visible colonies. After gently washing cells using PBS thrice, cells were subjected to 30 min of 4% paraformaldehyde fixation under ambient temperature followed by crystal violet staining. Colony images were made, and colony counting process was performed.

### 2.14. Migration Assays

Transwell assays were performed with chambers (pore size, 8 *μ*m; Corning Costar Corp, USA). Cells were cultured within the top chamber covered by serum-free medium, whereas the bottom chamber was added with 10% FBS-containing RPMI-1640 (500 *μ*l). After 24 h of incubation under 37°C, cells were subjected to 30 min of 4% paraformaldehyde fixation followed by another 30 min of crystal violet staining. Later, cells on the membrane surface were eliminated using cotton swabs. Cells on the membrane bottom surface were observed by using a microscope (Olympus) at a magnification of 10×.

### 2.15. Western Blot (WB) Analysis

The protein expression of vimentin, E-cadherin, N-cadherin, *β*-catenin, and GAPDH was determined by WB assay. The *β*-catenin (8480, 1 : 1000), vimentin (5741, 1 : 1000), N-cadherin (13116, 1 : 1000), E-cadherin (3195, 1 : 1000), and GAPDH (97166, 1 : 1000) antibodies were provided by Cell Signaling Technology (CST, Danvers, MA, USA).

### 2.16. Statistical Analysis

GraphPad (8.0) and *R* software (4.0) were utilized for statistical analysis. Differences in OS were evaluated by the log-rank test and KM analysis between low- and high-risk groups. Univariate together with multivariate Cox analysis was performed for identifying factors that independently predicted prognosis. Time-dependent receiver operating characteristic (t-ROC) curve analysis was carried out for evaluating the accuracy of our model in prognosis prediction. *p* < 0.05 stood for statistical significance.

## 3. Results

### 3.1. Determination of WGCNA Modules

**Figure 1:**
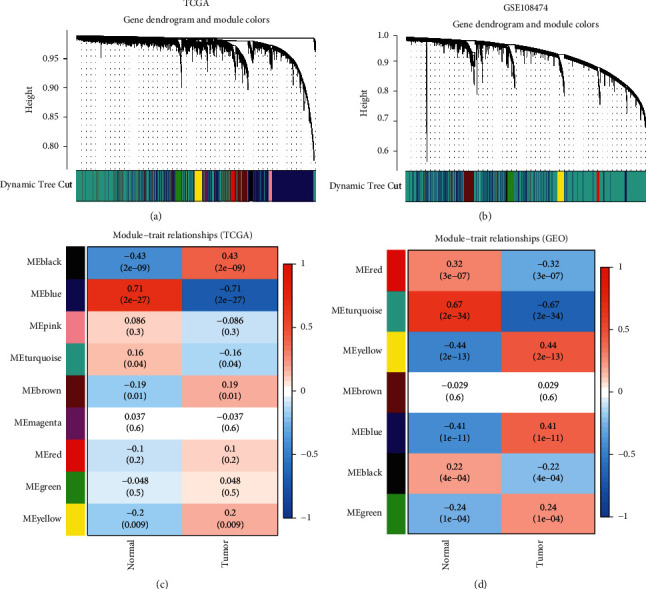
Determination of coexpression modules correlated with clinical status in the TCGA-GBM and GSE108474 data sets. (a) Coexpression module of TCGA-GBM. (b) Coexpression module of GSE108474. (c), (d) The relationship between coexpression module and clinical status in TCGA-GBM and GSE108474, respectively.

To explore the potential highly correlated modules, we first established gene coexpression networks based on GBM-TCGA and GSE108474 data sets (Figures 1(a) and 1(b)). By performing WGCNA, we obtained altogether 7 modules from GSE108474 and 9 from GBM-TCGA (the gray module not clustered to all clusters was excluded). Next, we analyzed the relationship between gene modules and clinical features (tumor and normal) by generated correlations heatmaps. As shown in Figures 1(c) and 1(d), turquoise and blue modules stood for modules significantly related to normal and glioma clinical traits.

### 3.2. Identification of Differential Coexpression Genes

**Figure 2:**
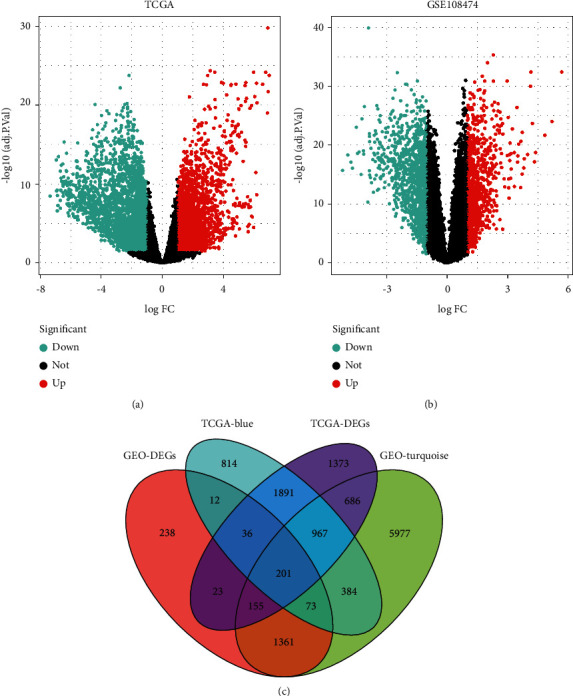
Detection of DEGs between GBM and normal tissues. (a) Volcano plot of DEGs in TCGA-GBM. (b) Volcano plot of DEGs in GSE108474. (c) Venn diagram indicating 201 differential coexpression genes.

We found that a total of 5289 DEGs and 2098 DEGs were screened out from the TCGA-GBM and the GSE108474 data sets, respectively (Figures 2(a) and 2(b)). Based on the most significantly related turquoise and blue modules observed from the heatmap above, we obtained 201 intersected genes for subsequent experiments (Figure 2(c)).

### 3.3. Construction of PPI Network

**Figure 3:**
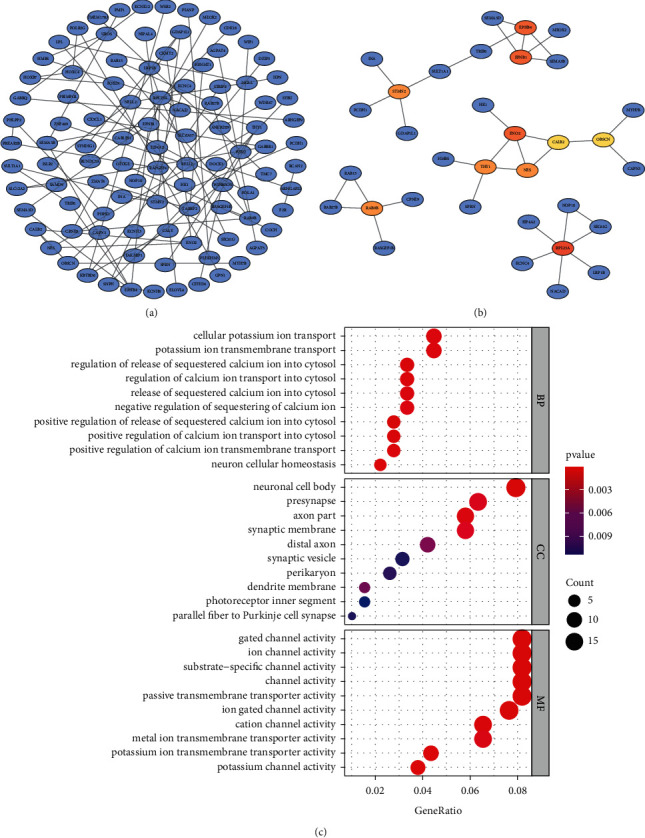
Construction of PPI network and functional enrichment. (a) PPI network of differential coexpression genes. (b) 10 hub genes of PPI network. (c) Gene ontology (GO) enrichment analysis.

The PPI network for those intersected genes was built based on the STRING database. After removing unconnected nodes, we mapped 81 edges and 201 nodes into the PPI network (Figure 3(a)). Furthermore, we detected hub genes from the network through MCC of Cytoscape. Those 10 hub genes identified included CALB2, EFNB1, ENO2, EPHB4, NES, OBSCN, RAB9B, RPL23A, STMN2, and THY1 (Figure 3(b)).

### 3.4. Functional Annotation for Overlapping Genes

To explore the biological functions of overlapping genes, we performed GO analysis, including BP, MF, and CC. According to Figure 3(c), GO analysis indicated significant enrichment in the BP of cell potassium ion transport, modulation of sequestered calcium ion production in cytosol, and potassium ion transmembrane transport. CC analysis suggested enrichment in the neuronal cell body, presynapse, and axon part. Additionally, the DEGs exhibited MF enrichment into substrate-specific channel activity.

### 3.5. Expression Patterns of Hub Genes

**Figure 4:**
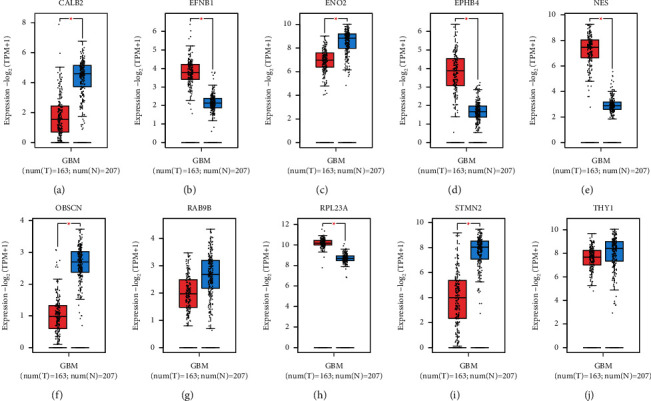
Detection of expression levels of the 10 hub genes between GBM samples and normal counterparts in GEPIA. (a) CALB2. (b) EFNB1. (c) ENO2. (d) EPHB4. (e) NES. (f) OBSCN. (g) RAB9B. (h) RPL23A. (i) STMN2. (j) THY1. Red represents cancer group, and blue represents normal group.

**Figure 5:**
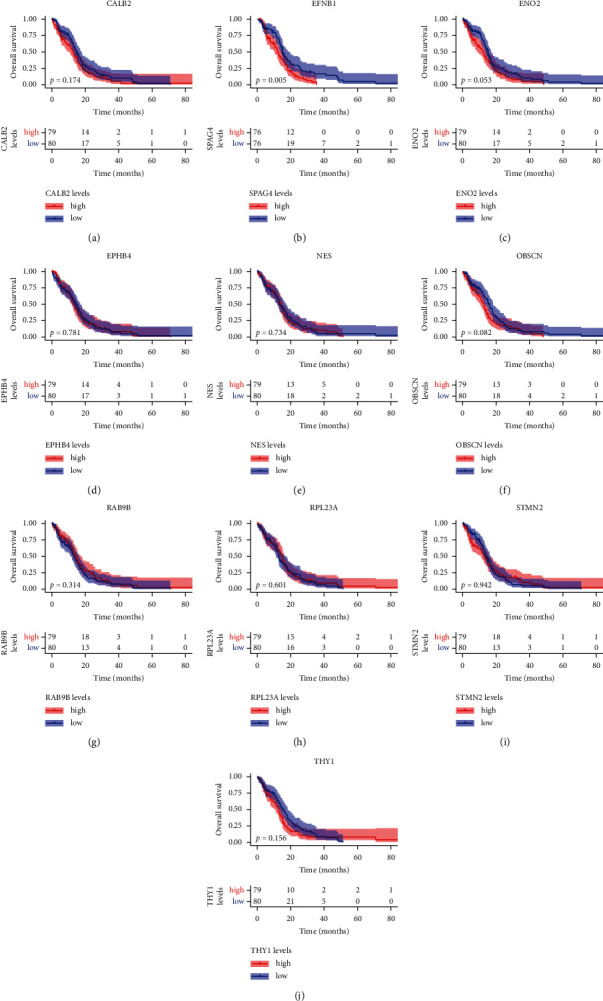
Kaplan–Meier curves for 10 hub genes. (a) CALB2. (b) EFNB1. (c) ENO2. (d) EPHB4. (e) NES. (f) OBSCN. (g) RAB9B. (h) RPL23A. (i) STMN2. (j) THY1.

We further validated 10 hub gene expressions within GBM and normal tissues by GEPIA online tool. Compared with normal tissues, RPL23A, EFNB1, NES, and EPHB4 expression markedly increased in GBM tissues, while CALB2, ENO2, OBSCN, RAB9B, and STMN2 were downregulated in GBM cases (Figures 4(a)–4(j)). According to KM curve analysis, only EFNB1 was dramatically correlated with poor clinical outcomes of the GBM cases (*p* < 0.05) (Figures 5(a)–5(j)). Therefore, we selected EFNB1 for the next analysis.

### 3.6. Association between EFNB1 and Immunomodulators

**Figure 6:**
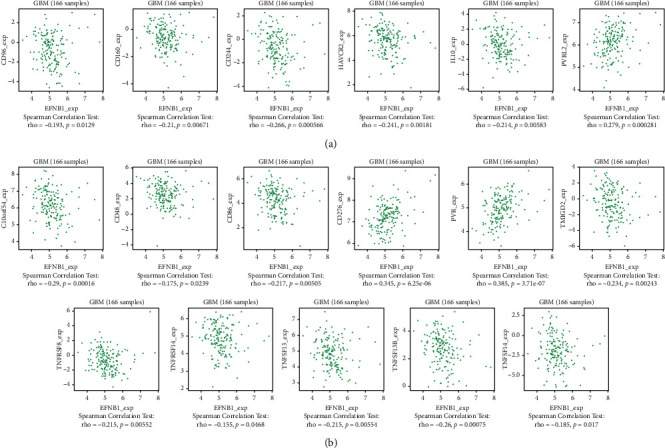
Correlation between EFNB1 expression levels and its immunomodulators. (a) EFNB1-related immunoinhibitors. (b) EFNB1-related immunostimulators.

To detect the role of EFNB1 on tumor-immune activity, we used the TISIDB online tool to obtain the EFNB1-associated immunomodulators. After using the TISIDB database, 6 immunoinhibitors (CD160, CD244, CD96, HAVCR2, IL10, and PVRL2) (Figure 6(a)) and 11 immunostimulators (C10orf54, CD276, CD48, CD86, PVR, TMIGD2, TNFRSF14, TNFRSF8, TNFSF13, TNFSF13B, and TNFSF14) (Figure 6(b)) were identified significantly associated with EFNB1 in GBM.

### 3.7. Identification of the Prognostic Value of EFNB1-Associated Immunomodulators in GBM

**Figure 7:**
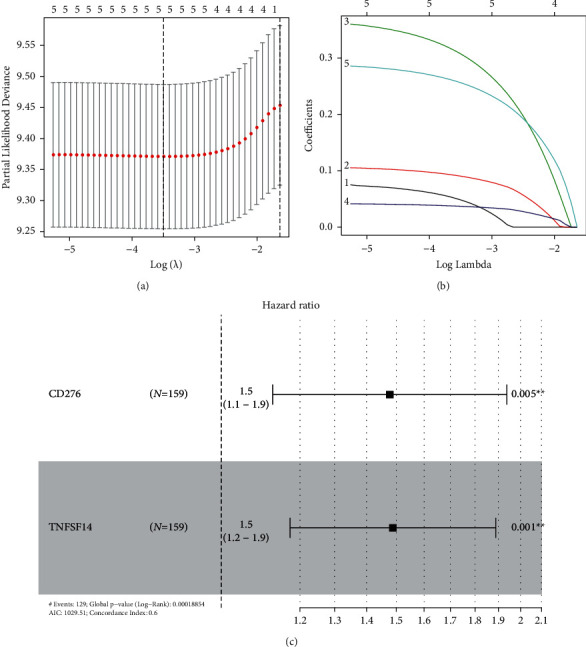
Construction of a risk model based on EFNB1-associated immunomodulators. (a) LASSO plot was created by the lambda sequence. (b) LASSO coefficient profiling of four genes. (c) Forest plot exhibiting the prognostic power of signature genes.

**Figure 8:**
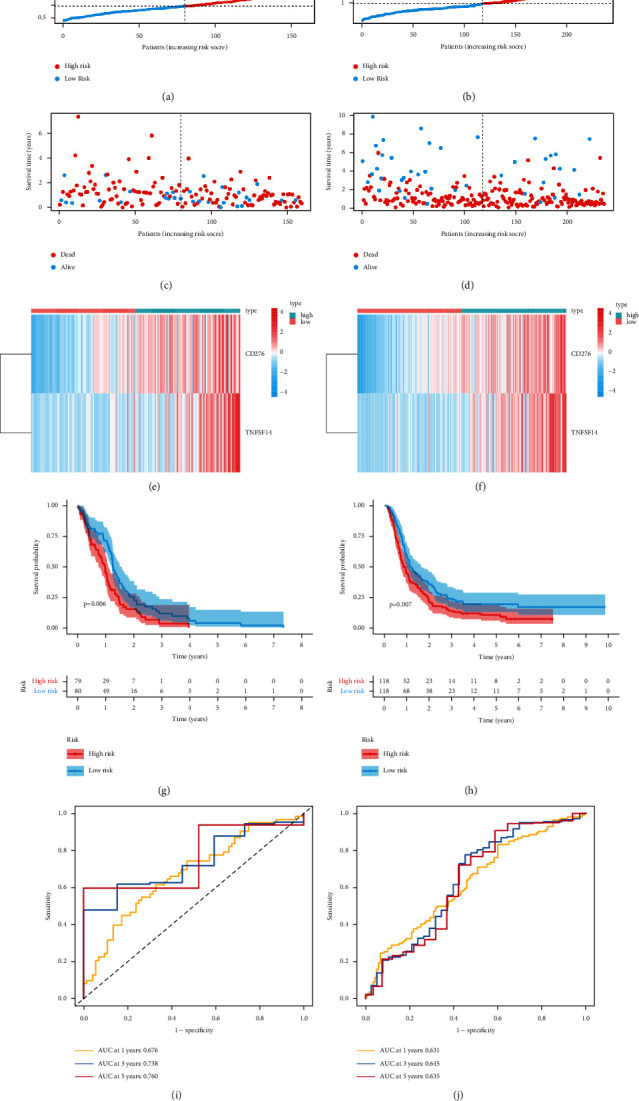
Prognostic performance of the risk signature. (a, b) Distribution of risk score in the TCGA and CGGA cohorts. (c, d) Survival outcomes of patients in the TCGA and CGGA data sets. (e, f) Heatmap displaying signature genes in the two risk groups. (g, h) Kaplan–Meier analysis of patients in the two risk groups. (i), (j) ROC curves at 1, 3, and 5 years in the TCGA and CGGA cohorts.

**Figure 9:**
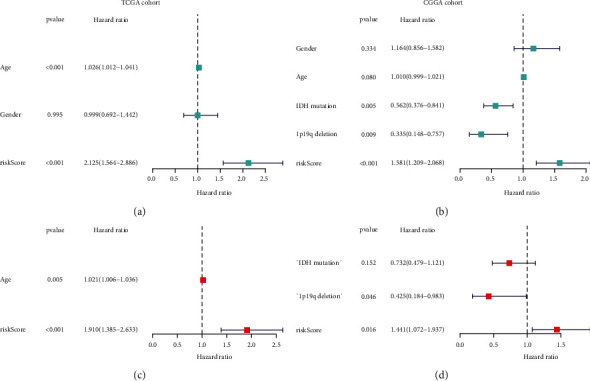
Univariate and multivariate Cox relative regressions of the risk score in the TCGA cohort (a, c) and CGGA cohort (b, d), respectively.

We further conducted Cox regression based on LASSO regression for constructing the prognosis prediction model by incorporating 17 EFNB1-associated immunomodulators in TCGA cohort (Figures 7(a) and 77(b)). A total of two risk genes (CD276 and TNFSF14) were identified for signature (Figure 7(c)). The formula is as follows: risk score = (0.3903 × CD276) + (0.3976 × TNFSF14). As revealed by K–M curves, high-risk patients had dismal clinical outcomes compared with low-risk patients. For verifying our model creditability, we conducted t-ROC curve analysis. In addition, the CGGA cohort proved our risk model constructed based on the training set (Figure 8). Univariate and multivariate Cox analyses were performed for investigating the independent prognostic ability of our risk signature. As revealed by results from the univariate regression, the risk score predicted OS of GBM. Furthermore, according to multivariate regression, our constructed risk score independently predicted prognosis of GBM (Figures 9(a) and 9(b)). Similar results were also validated in the CGGA cohort (Figures 9(c) and 9(d)).

### 3.8. Inhibition of EFNB1 Decreased GBM Cell Proliferation and Migration

**Figure 10:**
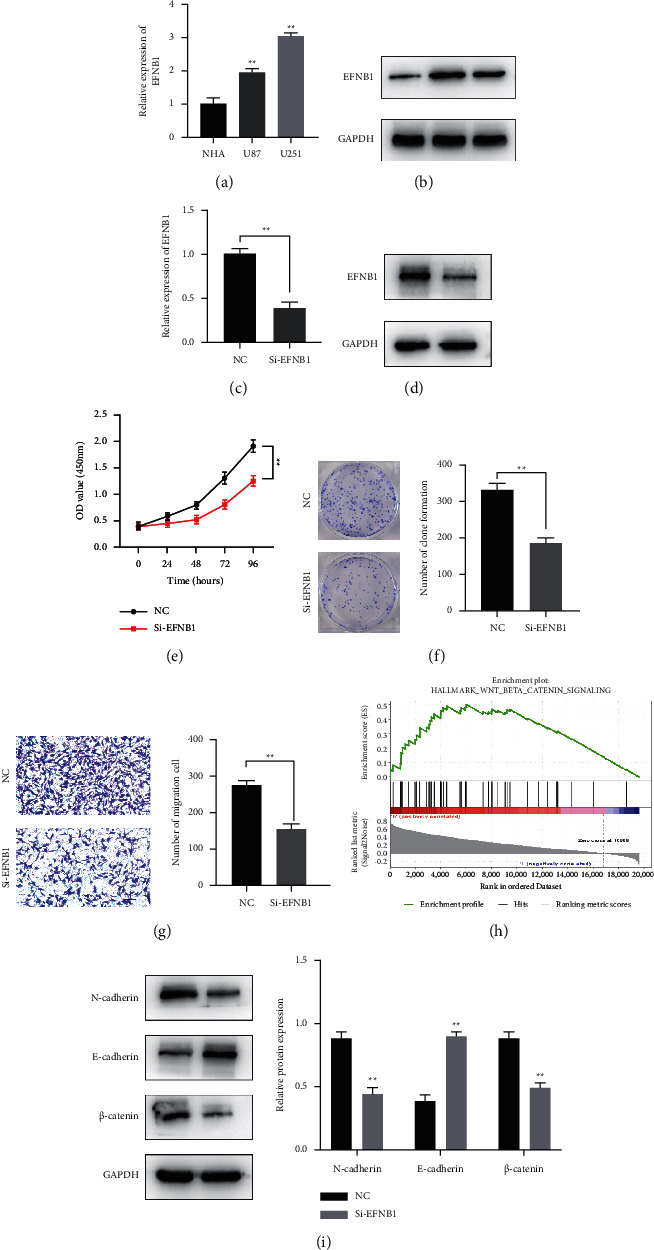
EFNB1 knockdown inhibited GBM proliferation and migration. (a, b) EFNB1 expression levels in the GBM cell lines were determined by qRT-PCR and western blot. (c, d) Silencing efficiency of EFNB1 in U251 cell. CCK-8 assays (e), clonogenic survival assay (f), and migration assay (g) were utilized to measure the rate after EFNB1 knockdown (scale bar = 100*μ*m). (h) GSEA analysis showed the Wnt/*β*-catenin pathway was enriched after EFNB1 overexpression. (i) Western blot was performed to detect the expression of E-cadherin, N-cadherin, *β*-catenin, and GAPDH in U251 cells with or without si-EFNB1 (^*∗*^*p* < 0.05; ^*∗∗*^*p* < 0.01; ^*∗∗∗*^*p* < 0.001).

To be started, we verified differential expression between GBM (U87, U251) and NHA cells by qRT-PCR and WB assays. As shown in Figures 10(a) and 10(b), EFNB1 was upregulated in GBM cells relative to NHA cells, especially in the U251 cell line. Next, we applied siRNAs for silencing EFNB1 within U251 cells and performed qRT-qPCR and WB to confirm its efficacy (Figures 10(c) and 10(d)). We found that the downregulation of EFNB1 expression dramatically suppressed U251 cell growth and migration, which was demonstrated in CCK8 proliferation assay, colony formation assay, and transwell assay (Figures 10(e)–10(g)). To detect the potential downstream pathways of EFNB1, GSEA suggested that the Wnt/*β*-catenin pathway was enriched after EFNB1 overexpression (Figure 10(h)). The protein expression of *β*-catenin, vimentin, and N-cadherin was markedly decreased in the si-EFNB1 group, while the opposite was observed for E-cadherin expression (Figure 10(i)). The above findings indicated the possible oncogenic role of EFNB1 in GBM through the Wnt/*β*-catenin pathway.

## 4. Discussion

Currently, more and more articles suggest that abnormal mRNA shearing is related to cancer migration, cell proliferation, apoptosis, angiogenesis, and metabolism [15–17]. Therefore, mRNA can be used as a biological marker to predict prognostic biomarkers of cancer. In this research, we discovered candidate biomarkers and examined the prognostic value by combining bioinformatics methods such as DEG screening, functional enrichment, TCGA data set verification, survival analysis, and PPI network establishment. Initially, we performed a systematic screening of differential mRNAs. GSE108474 and TCGA data sets were utilized to generate the gene coexpression network by WGCNA. Second, DEGs within cancer samples were selected from each of the two databases mentioned above. After that, we established the PPI network based on the intersected genes. Functional annotation analysis revealed a potential role of DEmRNAs in the pathogenesis of glioblastoma. GO analysis suggests that genes in this network were mainly involved in the regulatory mechanism of ion transport. Remodeling of calcium expression and activity regulates tumor growth and survival by participating in key cellular mechanisms and pathways, such as proliferation, migration, invasion, metastasis, and cell death [18]. Subsequently, we discovered 10 main hub genes from the PPI network, which included EFNB1, EPHB4, NES, RPL23A, CALB2, ENO2, OBSCN, RAB9B, STMN2, and THY1. Combined with survival and differential tissue expression analysis, EFNB1 upregulation predicted dismal survival of patients with primary glioblastoma.

The immune escape of cancer usually creates an immunosuppressive tumor microenvironment by recruiting suppressive cells and facilitating the depletion of immune cells, thus promoting tumor growth [19, 20]. Immunotherapy for patients with glioma remains suboptimal, which urgently requires the discovery of new immune checkpoints as therapeutic targets. In this study, we used the TISIDB online tool to obtain 17 EFNB1-related immunomodulators, including 6 immunosuppressive agents (CD160, CD244, CD96, HAVCR2, IL10, and PVRL2) and 11 immunostimulatory agents (C10orf54, CD276, CD48, CD86, PVR, TMIGD2, TNFRSF14, TNFRSF8, TNFSF13, TNFSF13B, and TNFSF14), whose functional analysis showed that they were relevant to EFNB1-mediated immune events, such as lymphocyte activation, leukocyte-cell adhesion, T-cell activation, and regulation of acquired immune responses. Lymphocytes are known to be an important part of the body's immune response [21, 22]. It includes natural killer cells, T-cells, and cytotoxic T lymphocytes, and all these are the critical factors related to the affected the cells- and tumor-targeting adaptive immune system [23, 24]. Leukocytes specifically express *β*2 integrin, which is a critical ingredient in the intercellular communication of immune cells, and perform an essential function in mediating intercellular adhesion and inhibiting immune activation [25, 26]. Next, we constructed a two-gene signature for GBM based on EFNB1-related immune modulators. Our constructed signature was highly accurate in the training and the validation data set. Based on clinical features, this study also developed an individualized predictive prognostic nomogram. This study might offer an accurate and simple approach for assessing GBM survival by clinicians.

In addition, we investigated the oncogenic role of EFNB1 in GBM cells by in vitro experiments. Compared with normal human astrocyte cells, EFNB1 expression was elevated in GBM cells. Inhibition of EFNB1 expression was able to significantly suppress the proliferation and migration of U251 cells. Knockdown of EFNB1 resulted in lower expression of *β*-catenin, vimentin, and N-cadherin and higher expression of E-cadherin, indicating that EFNB1 may have a positive regulatory effect on the Wnt signaling pathway. Hence, EFNB1 is likely to play a pro-carcinogenic role in GBM.

However, there are several shortcomings to our research. Our research is mostly based on bioinformatics methods. The expression pattern of EFNB1 needs to be verified in the local cohorts. Moreover, we will further explore the oncogenic effects of EFNB1 by *in vivo* experiments.

In summary, we proved that EFNB1 could act as a novel prognosis marker in GBM. Furthermore, a novel prognostic signature was developed based on EFNB1-related immunomodulators, which could be used as an independent predictor of prognosis and provide new clues for immunotherapy in GBM patients.

## Data Availability

Publicly available data sets were analyzed in this study. These data can be found at the TCGA (https://tcga-data.nci.nih.gov/tcga/), GEO (http://www.ncbi.nlm.nih.gov/geo/), CGGA database (http://www.cgga.org.cn/), and TISIDB databases (http://cis.hku.hk/TISIDB/).

## Supplementary Materials

Supplementary Materials Supplementary Table 1: target sequence of si-NC and si-EFNB1. Supplementary Table 2: primers utilized for qRT-PCR.
