# Supplementary material for: EFNB1 Acts as a Novel Prognosis Marker in Glioblastoma through Bioinformatics Methods and Experimental Validation
Source: J Oncol. 2021 Nov 16;2021:4701680. doi: 10.1155/2021/4701680 (PMC8610726; doi:10.1155/2021/4701680)
Supplement: Supplementary Materials — Supplementary Table 1: target sequence of si-NC and si-EFNB1. Supplementary Table 2: primers utilized for qRT-PCR. [file 4701680.f1.zip › 4701680.f1/Supplementary Table 1.doc]

**Supplementary Table 1. Target sequence of si-NC and si-EFNB1**

| Gene | Target sequence |
| --- | --- |
| si-EFNB1 | 5’-TGACGGTCCTACTACTGAA-3’ |
| si-NC | 5’-UUCUCCGAACGUGUCAGGUTT-3’ |
