# Supplementary material for: EFNB1 Acts as a Novel Prognosis Marker in Glioblastoma through Bioinformatics Methods and Experimental Validation
Source: J Oncol. 2021 Nov 16;2021:4701680. doi: 10.1155/2021/4701680 (PMC8610726; doi:10.1155/2021/4701680)
Supplement: Supplementary Materials — Supplementary Table 1: target sequence of si-NC and si-EFNB1. Supplementary Table 2: primers utilized for qRT-PCR. [file 4701680.f1.zip › 4701680.f1/Supplementary Table 2.doc]

**Supplementary Table 2. Primers utilized for qRT-PCR**

| Gene | 5'to 3' |
| --- | --- |
| EFNB1 | Forward CAGCCGGCCAAGCAAAGAGT |
|  | Reverse GGGGAGAAAAGAGAGGCCAA |
| GAPDH | Forward ACAACCTTTGGTATCGTGGAAGG |
|  | Reverse GCCATCACGCCACAGTTTC |
